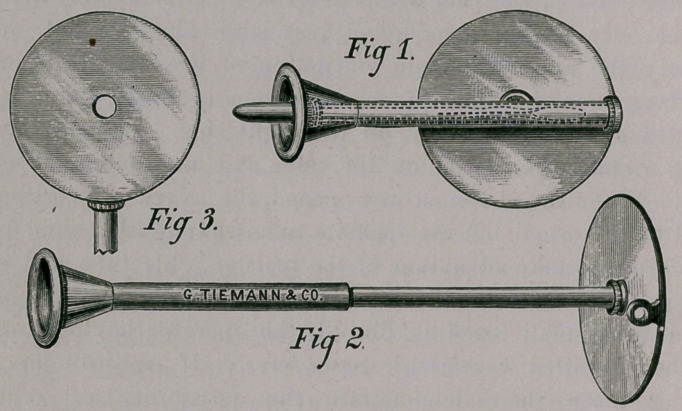# The Polyscope

**Published:** 1890-08

**Authors:** Ernest Wende

**Affiliations:** Professor of Dermatology in the University of Buffalo; 174 Franklin Street


A NEW INSTRUMENT, BEING A COMBINATION OF STETHOSCOPE, THERMOSCOPE, LARYNGOSCOPE, AND OTOSCOPE. DEVISED BY ERNEST WENDE, M. D., B. SC., PROFESSOR OF DERMATOLOGY IN THE UNIVERSITY OF BUFFALO.

Fig 1. Fig 3. Fig 2.

The accompanying cuts represent an instrument which may prove of some diagnostic value. It combines stethoscope thermometer and reflector, so constructed that it can be readily packed in a small compass and carried in the upper vest pocket. It consists of an ordinary single-barreled stethoscope, so designed that the ear-piece, which is made of hard metal and highly polished, can be quickly adjusted and employed as a reflector for the examination of nose, throat, or ear. The reflector has a focal distance of about six inches. The reflecting efficiency of its surface being equal to that of the ordinary mirror used for that purpose.

Fig. 1, represents the instrument closed, the barrel being telescoped and reflector lying flat upon it. The dotted lines denote the position of thermometer securely held by means of a metallic cap and screw, rendering breakage impossible.

Fig. 2, shows stethoscope adjusted for use.

Fig. 3, shows reflector in position with barrel of stethoscope to be used as handle. The advantages obtained are these — durability, portability, its various applications, and readiness of manipulation.

The instrument was most admirably made for me by G. Tiemann & Co., 107 Park Row, New York.

174 Franklin Street.

## Figures and Tables

**Fig 1. Fig 2. Fig 3. f1:**